# Seed response to strigolactone is controlled by abscisic acid-independent DNA methylation in the obligate root parasitic plant, *Phelipanche ramosa* L. Pomel

**DOI:** 10.1093/jxb/erv119

**Published:** 2015-03-28

**Authors:** Marc-Marie Lechat, Guillaume Brun, Grégory Montiel, Christophe Véronési, Philippe Simier, Séverine Thoiron, Jean-Bernard Pouvreau, Philippe Delavault

**Affiliations:** Laboratoire de Biologie et Pathologie Végétales, SFR 4207 QUASAV, Université de Nantes, 2 rue de la Houssinière, BP 92208, 44322 Nantes Cedex 3, France

**Keywords:** Bisulfite sequencing, branched broomrape (*Phelipanche ramosa*), DNA methylation, dormancy release, epigenetic regulation, parasitic plant, seed germination, strigolactone.

## Abstract

Seed dormancy release of the obligate root parasitic plant, *Phelipanche ramosa*, requires a minimum 4-day conditioning period followed by stimulation by host-derived germination stimulants, such as strigolactones. Germination is then mediated by germination stimulant-dependent activation of *PrCYP707A1*, an abscisic acid catabolic gene. The molecular mechanisms occurring during the conditioning period that silence *PrCYP707A1* expression and regulate germination stimulant response are almost unknown. Here, global DNA methylation quantification associated with pharmacological approaches and cytosine methylation analysis of the *PrCYP707A1* promoter were used to investigate the modulation and possible role of DNA methylation during the conditioning period and in the *PrCYP707A1* response to GR24, a synthetic strigolactone analogue. Active global DNA demethylation occurs during the conditioning period and is required for *PrCYP707A1* activation by GR24 and for subsequent seed germination. Treatment with 5-azacytidine, a DNA-hypomethylating molecule, reduces the length of the conditioning period. Conversely, hydroxyurea, a hypermethylating agent, inhibits *PrCYP707A1* expression and seed germination. Methylated DNA immunoprecipitation followed by PCR experiments and bisulfite sequencing revealed that DNA demethylation particularly impacts a 78-nucleotide sequence in the *PrCYP707A1* promoter. The results here demonstrate that the DNA methylation status during the conditioning period plays a crucial role independently of abscisic acid in the regulation of *P. ramosa* seed germination by controlling the strigolactone-dependent expression of *PrCYP707A1*.

## Highlight

This study demonstrates for the first time that DNA demethylation, an epigenetic mechanism, can control parasitic plant seed response to the strigolactones, a new class of plant hormone.

## Introduction

Some parasitic plants are deadly pests with the capacity to exploit other plants. Among the obligate root parasitic weeds, the holoparasites, which are devoid of chlorophyll and thus unable to carry out photosynthesis, totally rely on a host for their water, mineral, and carbohydrate supplies. Members of the genus *Orobanche* and *Phelipanche*, belonging to the Orobanchaceae family, are the final result of an evolutionary transition from autotrophism to heterotrophism (Westwood *et al.*, 2010). The underlying process of this trophic exploitation, governed by a fine-tuned molecular dialogue between both partners, is an extraordinary example of adaptive plant biology operated by these parasitic organisms in the course of evolution. Among the remarkable morphological and physiological adaptations that characterize this transition, the requirement for the seeds to perceive molecules produced by host roots to germinate is probably the most intriguing one. Indeed, the germination of broomrape species is a two-step process corresponding first to a conditioning period, thought to be required for the acquisition of the sensitivity to germination stimulants (GS) (Joel *et al.*, 1991), followed by the chemical stimulation of the germination itself that ends with the radicle protrusion (Joel and Bar, 2013).

During the conditioning period, broomrape seeds are exposed to a moist environment and suitable temperatures that result in physiological processes that set up the machinery needed for GS perception. In *Phelipanche ramosa*, seeds require a minimum of 4 days of conditioning at 21°C to allow optimal germination in response to GS (Lechat *et al.*, 2012). The conditioning period starts with water entry into the seed through the micropyle, which opens after 30min (Joel *et al.*, 2012) and results in seed imbibition that takes around 1h (Lechat *et al.*, 2012). Several physiological processes then occur that indicate a rapid metabolic reactivation. First, an optimal adenylate energy charge is reached on the first day of conditioning, concomitant to a strong decrease in abscisic acid (ABA) seed content due to an important release into the medium (Chae *et al.*, 2004; Lechat *et al.*, 2012). Protein synthesis, utilization of reducing sugars, and a characteristic pattern of respiration with a strong alternative oxidase activity are also observed (Bar-Nun and Mayer, 1993; 2002; Bar-Nun *et al.*, 2003). Although the exact underlying mechanisms are not yet known, all these events are potentially involved in the set-up of the mechanisms needed for GS perception or signal transduction and therefore for seed germination in response to GS. Once conditioned, broomrape seeds are still unable to germinate without stimulation by GS, chemical compounds produced and exuded in the rhizosphere by surrounding host roots.

While the key role of GS, such as strigolactone (SL), has been known for several decades, until recently almost nothing was known about the early molecular events governing GS-induced seed germination in root parasitic plants. In 2012, Lechat *et al*. highlighted the major role of *PrCYP707A1*, an ABA catabolic gene, in *P. ramosa* seed germination in response to the synthetic SL analogue, GR24. GR24 treatment of conditioned seeds triggered a rapid and strong up-regulation of *PrCYP707A1* during the first 18h, followed by an 8-fold decrease in ABA levels detectable after 3 days of treatment. The concomitant application of ABA or Abz-E2B, a specific inhibitor of CYP707A enzymes (Okazaki *et al.*, 2012), prevented germination of conditioned seeds in the presence of GR24 (Lechat *et al.*, 2012; Pouvreau *et al.*, 2013). These results demonstrated that germination occurs after a dormancy release of the conditioned seeds triggered by ABA catabolism and mediated by the GR24-dependent activation of the *PrCYP707A1* gene. Interestingly, it has also been determined that *PrCYP707A1* expression cannot be triggered by GR24 during a minimal 4-day period following imbibition. This indicates that seeds remain insensitive to SL during this period through a repressive mechanism that must be overcome to allow seed germination. Altogether, these observations suggest that the conditioning period can be defined as the minimal period required for broomrape seeds to gain the ability to germinate in response to SL, which can be monitored through the induction of *PrCYP707A1* expression. It is therefore thought that sensitivity to SL is acquired during this conditioning phase (Matusova *et al.*, 2004), which would be similar to the stratification process of non-parasitic plants (Gubler *et al.*, 2005).

Although the existence of a seed germination lag phase cannot be denied, the molecular mechanisms governing its relief remain unknown. In general, seed dormancy and germination are known to be hormone-dependent (mainly ABA and gibberellins) and involve up-regulation or silencing of several associated genes (Finkelstein *et al.*, 2008). Within the root parasitic plants, a strong decrease of ABA content naturally occurs during the conditioning period in *Orobanche minor* and *P. ramosa* seeds, followed by a second decrease attributed to GS-induced PrCYP707A1 activity (Chae *et al.*, 2004; Lechat *et al.*, 2012). Thus, it might be suggested that the conditioning period is controlled by endogenous ABA levels.

Plants respond to environmental cues by epigenetic mechanisms (Grimanelli and Roudier, 2013), including the modification of DNA methylation status (Richards, 1997). Concerning DNA methylation, promoters or coding regions of silent genes are, for instance, found to be more methylated than actively transcribed sequences (Finnegan *et al.*, 1993), indicating that DNA methylation can regulate gene expression (Martienssen and Colot, 2001; Chan *et al.*, 2005). In mammals, DNA methylation only affects the symmetrical CG sequences by conversion of cytosine to 5-methylcytosine (5-mC) (Bird, 2002); in plants, CG, CHG, CNG, and CHH motifs are affected (Gruenbaum *et al.*, 1981; Vanyushin, 2006). Cytosine methylation thus plays essential roles in the regulation of plant gene expression in a developmental stage-dependent or tissue-specific manner, notably during seed development (Chan *et al.*, 2005; Gehring and Henikoff, 2007; Kapazoglou *et al.*, 2012). Indeed, dormancy release and germination are accompanied by cytosine demethylation in the seeds of oilseed rape (*Brassica napus*) (Lu *et al.*, 2006), pepper (*Capsicum annuum L.*) (Portis *et al.*, 2004), and wheat (*Triticum aestivum* L.) (Meng *et al.*, 2012).

In the present study, ABA implication and DNA methylation modulation were investigated during the conditioning period of seeds of the parasitic plant, *P. ramosa*. The findings demonstrate that cytosine demethylation occurring during the conditioning period is a key step in seed germination by controlling the *PrCYP707A1* response to SL.

## Materials and methods

### Plant material

*P. ramosa* (L.) Pomel seeds were collected in 2012 from mature flowering spikes of broomrape plants parasitizing oilseed rape (*Brassica napus*; Saint-Jean-d’Angély, France), and were stored at 25°C in darkness until use. Seeds were surface-sterilized according to Lechat *et al.* (2012): 5min in sodium hypochlorite (2.4%), and thoroughly rinsed three times for 1min and three times for 5min with sterile distilled water. Seeds were then suspended in incubation medium (IM, 1mM Na/K phosphate buffer; pH7.5, adjusted with KOH) and 0.1% Plant Preservative Mixture (Kalys, Bernin, France), with a ratio of 10mg seed mL^−1^ for germination assay or 40mg seed mL^−1^ for sample preparation. Seeds were then placed in the dark at 21°C during the conditioning period.

### Germination assay

Sterilized seeds were conditioned from 0 to 7 days in a 96-well plate (Cell Culture Multiwell Plate Cellstar; Greiner Bio-One, Frickenhausen, Germany) at 21°C and in darkness according to Pouvreau *et al.* (2013) in IM supplemented or not with 250 µM 5-azacytidine, a hypomethylating agent (Sigma-Aldrich, St. Louis, MO, USA), 250 µM hydroxyurea, a hypermethylating agent (Sigma-Aldrich), or 250 µM ABA, in a final volume of 100 µL per well. Tested molecules were added to the IM from the first day of the conditioning period and maintained throughout the experiment even after GR24 treatment. Following the different conditioning periods, 1nM GR24 (0.1% acetone, v/v) was added to the IM. An IM supplemented with 0.1% acetone (v/v) was used as a negative control. Subsequently, plates were incubated for 3 days at 21°C in the dark, and germination percentages were determined using methylthiazolyldiphenyl-tetrazolium bromide as previously described (Pouvreau *et al.*, 2013). Seeds were considered to be germinated when the radicle protruded out of the seed coat. Germination assays were repeated at least six times. For seed viability tests following hydroxyurea or ABA application during the conditioning period, treated seeds were washed three times with 100 μL of IM before adding 100 μL of IM with 1nM GR24 in 0.1% acetone. Plates were incubated as mentioned above prior to determining germination percentage.

### Sample preparation

For molecular analyses, i.e. gene expression and DNA methylation, 5mL of sterilized *P. ramosa* seeds (200mg) were conditioned in the dark at 21°C in tissue culture flasks (Becton Dickinson, Franklin Lakes, NJ, USA) in IM, in IM supplemented with 1mM 5-azacytidine or 1mM hydroxyurea, or in 1mM ABA. Seeds were collected each day for 7 days by filtration on 100 µm nylon mesh, blotted on absorbent paper, then frozen in liquid nitrogen and stored at −80°C before subsequent nucleic acid purification. To determine *PrCYP707A1* expression, seeds were stimulated with 1nM GR24 in 0.1% acetone for 6h before being collected. For all experiments, a fresh aliquot was conserved to evaluate the germination rate after chemical treatment, according to the protocol established by Pouvreau *et al.* (2013).

### DNA and RNA extraction

Total DNA was isolated from 200mg of treated seeds using Nucleospin Plant II (Macherey Nagel, Hoerdt, France), according to manufacturer’s instructions. DNA was quantified using spectrophotometry (A_260_/A_280_; NanoDrop Spectrophotometer ND-1000, Labtech International Ltd, Rigmer, UK) and stored at −20°C before use.

Total RNA was isolated from 200mg of 6h GR24-treated seeds, using the Nucleospin RNA Plant kit (Macherey Nagel) according to manufacturer’s instructions, with specific lysis buffer guanidinium-HCl (RAP buffer). RNA extracts were then treated with 6.8 Kunitz units of RNase-Free DNase I (Qiagen, Courtaboeuf, France), cleaned, and concentrated with RNA clean up XS kit (Macherey Nagel). The integrity of total RNA was checked by electrophoresis on a 2% (w/v) agarose gel and RNA extracts were quantified with spectrophotometry (A_260_/A_280_; NanoDrop Spectrophotometer ND-1000).

### Real-time reverse transcription polymerase chain reaction

cDNA was synthesized from 0.5 μg of total RNA using the Superscript II Reverse Transcriptase (Invitrogen, Carlsbad, CA, USA) following the manufacturer’s instructions. RT-PCR experiments using SYBR Green technology were carried out on an Applied Biosystems 7300 real-time PCR system (Applied Biosystems, Carlsbad, CA, USA) according to Lechat *et al.* (2012). Specificity of the PCR amplification was checked using a heat dissociation protocol (from 60°C to 95°C) after the final cycle of PCR. Fold change in RNA expression was estimated using threshold cycles. The amplicon of the constitutive elongation factor *PrEF1α* (Table 1), which showed low cycle threshold variation (SD <0.5 cycle threshold), was used as an internal control to normalize all the data (Péron *et al.*, 2012). The gene-specific primers used for each amplification are presented in Table 1. A control experiment without cDNA was included for each PCR mix. Three biological replicates were performed, each in three technical replicates. An analysis of variance was performed on the results from RT-PCR analyses using SigmaPlot version 10.0. Means of three independent RNA isolations were tested at *P* < 0.05 (Tukey test).

**Table 1.** Primers used in the present study

Gene	Primer name	Forward and reverse primers 5ʹ→3ʹ	Product size (bp)	Application
*PrEF1-a*	*Q-PrEF1-a-F*	TTGCCGTGAAGGATCTGAAAC	63	RT-PCR control
	*Q-PrEF1-a-R*	CCTTGGCAGGGTCGTCTTTA		
*PrCYP707A1*	*Q-PrCYP707A1-F*	GCCCGCTCTCAAAAGCTAAA	60	RT-PCR
	*Q-PrCYP707A1-R*	TTGTAACAGATTTGGGCTTTTGG		
*PrCYP707A1*	*GWPrCYP707A1-R*	CCTGGTGGGAGAGGCAGTTTTACATGG	2581	Chromosome walking
	*Ap1*	GTAATACGACTCACTATAGGGC		
*PrCYP707A1*	*MeDIP −2117/−2010-F*	TAGGCCCAAAGAGTCCAAAATCC	107	Quantitative PCR MeDIP
	*MeDIP −2117/−2010-R*	TGGAACCTCATCCTGCCTATC		
*PrCYP707A1*	*MeDIP −2027/−1831-F*	GGCAGGATGAGGTTCCAAAAC	196	Quantitative PCR MeDIP
	*MeDIP −2027/−1831-R*	GCCAAATGAATCTAAAACGAGAC		
*PrCYP707A1*	*MeDIP −1851/−1753-F*	TCGTTTTAGATTCATTTGGCTCTA	98	Quantitative PCR MeDIP/ MS-PCR
	*MeDIP −1851/−1753-R*	CATATTTTGCATGTTTACGATTTGTC		
*PrCYP707A1*	*MeDIP −679/−570-F*	GACCGAAGAAGGTCCGTATTTAT	109	Quantitative PCR MeDIP
	*MeDIP −679/−570-R*	CTGCTGTCTCAACCGATCATATG		
*PrCYP707A1*	*MeDIP −549/−478-F*	TGACGGAAGTGAGAGAGCAGTT	71	Quantitative PCR MeDIP
	*MeDIP −549/−478-R*	CCGAGACATGATCTGCAGTACA		
*PrCYP707A1*	*MeDIP −529/−447-F*	TTTCAGATGAAGTCGCAGATGT	82	Quantitative PCR MeDIP
	*MeDIP −529/−447-R*	GCTATCCATGCTCCAGTATATCCT		
*PrCYP707A1*	*Bisulfite −2181/−1728-F*	TTTAAGGGGGGGTATTTATAGTT	453	Bisulfite sequencing/MS−PCR
	*Bisulfite −2181/−1728-R*	TATCTCRTTTCRTATTAATTAAACTCA		
*PrCYP707A1*	*McrBC −2130/−1830-R*	CCTAAGGGGGGGTATTTATAGCT	300	MS-PCR
	*McrBC −2130/−1830-R*	TGAGCTCAATTAACACGAAACGAGACA		

### Determination of *PrCYP707A1* promoter sequence

The sequence of the *PrCYP707A1* promoter was obtained using the GenomeWalker Universal Kit (Clontech, Palo Alto, CA, USA) according to manufacturer’s instructions. The promoter was amplified by a single PCR reaction using specific adaptor primers AP1 and a gene-specific primer GWPrCYP707A1 (Table 1). A single PCR product was cloned using a pGEM-T Easy Vector System kit (Promega, Madison, WI, USA) according to manufacturer’s instructions, and then sequenced (Eurofins MWG Operon, Ebersberg, Germany). A *PrCYP707A1* promoter sequence of 2581bp was obtained and assembled with the *PrCYP707A1* gene sequence (GenBank accession number: KM226163). Promoter motif searches were carried out using the PLACE software (Higo *et al.*, 1999).

### Global DNA methylation quantification

Global DNA methylation quantification was conducted using the MethylFlash Methylated DNA Quantification Kit according to manufacturer’s instructions (Epigentek Inc., Farmingdale, NY, USA). The protocol allowed the colorimetric quantification of global DNA methylation by specifically measuring levels of 5-mC in an ELISA-like microplate-based format. Three biological replicates, were performed, with three technical repetitions each. DNA (100ng) was bound to strip wells that were specifically treated to have a high DNA affinity. The methylated DNA fraction was detected using capture and detection antibodies and then quantified using colorimetry at 450nm in a POLARstar Omega microplate spectrophotometer (BMG Labtech, Ortenberg, Germany). Methylated DNA amount was proportional to the OD. The absolute amount of methylated DNA was quantified from a standard curve. The quantity and percentage of 5-mC, referred to as methylated DNA, in total DNA were calculated using the following formulas:

$$\text{5-mc(ng)=}\frac{\text{Sample OD-ME3 OD}}{\text{Slope}\times \text{2}}$$ (1)

$$\text{5-mC(\%)=}\frac{\text{5-mC(ng)}}{\text{S}}\times \text{100}$$ (2)

S is the amount of input sample DNA (ng). ME3 is the negative control. The slope of the standard curve was obtained after linear regression.

### Methylation analysis of *PrCYP707A1* promoter

The *PrCYP707A1* promoter sequence was investigated with MethPrimer software (http://www.urogene.org/cgi-bin/methprimer/methprimer.cgi) to determine the presence of CpG islands using the following identification parameters: Island size > 100bp, GC% > 50, and Obs/Exp > 0.6. Methylation levels of the *PrCYP707A1* promoter during the conditioning period were quantified using a methylated DNA immunoprecipitation (MeDIP)-PCR kit according to manufacturer’s instructions (ActiveMotif Europe, La Hulpe, Belgium). Genomic DNA was extracted from seeds conditioned for 0 and 7 days and sonicated for 15 cycles of 40 pulses (30% amplitude) to generate random fragments of ±500bp using a sonicator Sonifier S-450A (Brandon Ultrasonics Corporation, Danbury, CO, USA). Sonicated DNA fragments were divided in two parts: one was used as input DNA (normalized control) and the other was prepared for MeDIP. For each immunoprecipitation (IP) assay, 500ng of fragmented DNA was used. After denaturation at 95°C for 10min, samples containing single-stranded DNA were chilled on ice for 10min. IP was performed using 2 µg monoclonal antibody targeting 5-mC residues and 2 µg bridging antibody in a final volume of 100 µL. To capture complexes of 5-mC residues, 25 µL Protein G magnetic beads were added for 12h at 4°C. The magnetic beads were then washed with appropriate buffers and IP DNA fragments were eluted in a final volume of 100 µL according to manufacturer’s instructions. A treatment with 1 µg of Proteinase K was performed at 50°C for 30min following by inactivation at 80°C for 10min. Treated DNA was then purified according to manufacturer’s instructions. Real-time PCR was performed to amplify 5 µL of each purified single-stranded DNA sample using specific primer sets designed with Primer Express 3 software. For each CpG island, three primer sets were designed: MeDIP −679/−570, MeDIP −549/−478, and MeDIP −529/−447 for CpG island 1; and MeDIP −2117/−2010, MeDIP −2027/−1831, and MeDIP −1851/−1753 for CpG island 2 (Table 1). For each primer set and each conditioning time point, a standard amplification curve was determined using input DNA (5, 0.5, 0.05, and 0.005ng/µL) to determine the amount of immunoprecipitated DNA. The percentage between the immunoprecipitated DNA and the initial quantity of DNA used for IP (500ng) was then calculated (% of input DNA).

### Restriction analysis and methyl-sensitive PCR semi-quantitative amplification

Methyl-sensitive PCR (MS-PCR) was performed using a methyl-sensitive restriction enzyme. Three replicates of 500ng of total DNA, extracted from seeds conditioned for 0 (D0), 3 (D3), 4 (D4), and 7 (D7) days, were digested with 5U of *McrBC* (New England BioLabs, Ipswich, UK) overnight at 37°C. *McrBC* recognizes DNA containing two methylated cytosine residues (A/G 5-mC) separated by 40–2000bp and cleaves the DNA at multiple sites close to one of the methylated sites. Digested and undigested total DNA was analysed by electrophoresis on a 1% agarose gel, purified using Nucleospin Plant II (Macherey Nagel), and quantified using spectrophotometry (A260/280; 260/230; NanoDrop Spectrophotometer ND-1000, Labtech International Ltd). Digested or undigested DNA (1ng) was amplified by PCR using specific primers (Bisulfite −2181/−1728; McrBC −2130/−1830, and MeDIP −1851/−1753; Table 1) and Q5 high fidelity polymerase (New England BioLabs). After denaturation at 98°C for 30 s, amplification consisted of 30–40 cycles of 10 s at 98°C, 30 s at 55°C, and 20 s at 72°C. The number of PCR cycles was adjusted to avoid a saturation of amplification. PCR products were analysed by electrophoresis on 1% agarose gel.

### Bisulfite sequencing

The DNA methylation status at the nucleotide level of the *PrCYP707A1* 5ʹ-upstream region was investigated using bisulfite sequencing. Because bisulfite treatment converts unmethylated cytosines to uracils, it was used to determine the methylation status of cytosines in CG, CHG, and CHH contexts (where H could be A, T, or C). Total DNA, extracted from D0 and D7 seeds, were treated with bisulfite using the Zymo Research EZ DNA Methylation-Gold Kit (Irvine, CA, USA). DNA (600ng) of three biological replicates for each condition was converted using the most efficient conditions described in literature: 95°C for 4min and 47°C for 1.5h (Kiselev *et al.*, 2013). A primer set, Bisulfite −2181/−1728 (Table 1), was designed by Methyl Primer Express Software v1.0 to amplify the whole CpG island 2 localized between −2183 and −1708bp upstream of the transcription start site (−73bp from ATG). PCR products were cloned using the pGEM-T Easy Vector Systems kit (Promega) and a total of 20 clones per condition were sequenced (Eurofins MWG Operon). For a given cytosine, the cytosine methylation variation expressed as a percentage was calculated according to the following formula: (number of clones showing a methylated cytosine at D7 minus the number of clones showing a methylated cytosine at D0) divided by total clone number. Moreover, following Trap-Gentil *et al.* (2011), when more than 50% of clones exhibited a methylated cytosine in any position, this cytosine was considered as hypermethylated.

## Results

### ABA and expression of the GR24 responsive gene *PrCYP707A1*

**Fig. 1.**
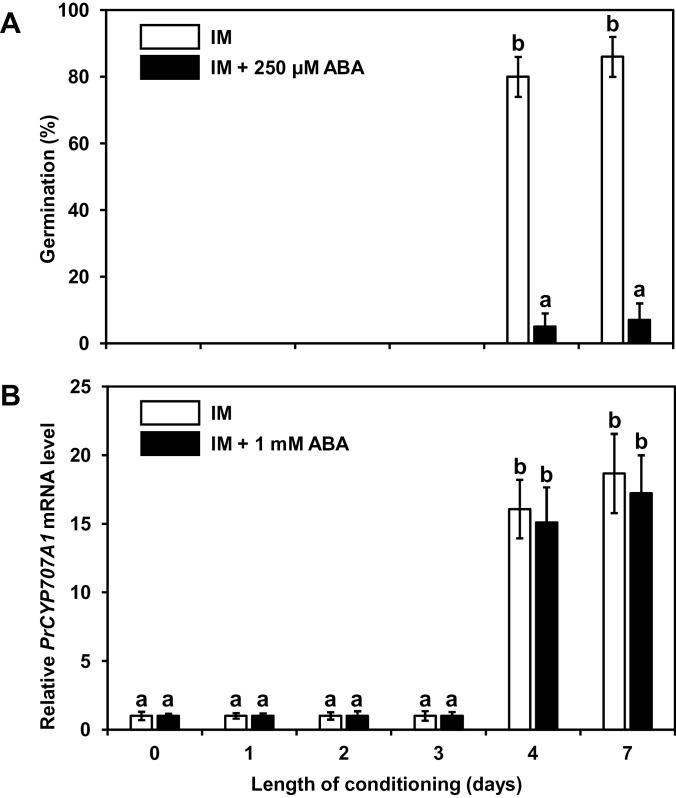
The conditioning period is not controlled by ABA. **(A)** Germination rate of *P. ramosa* seeds conditioned in IM supplemented or not with 250 µM ABA. After different lengths of conditioning, seeds were stimulated by 1nM GR24 and germination rates were determined after 3 days. Means are values ± SD (n = 6). **(B)** RT-PCR analysis of the *PrCYP707A1* expression in seeds conditioned for different lengths of conditioning in IM supplemented or not with 1mM ABA. After conditioning, seeds were stimulated by 1nM GR24 and expression levels were determined after 6h. Means are values ± SD (n = 3). Means with the same letter are not significantly different from each other (Tukey test, *P* < 0.05).

Because ABA was shown to inhibit seed germination in *P. ramosa* (Pouvreau *et al.*, 2013) and was suggested to control the acquisition of sensitivity to GS during the conditioning period (Lechat *et al.*, 2012), seeds were conditioned in IM supplemented or not with ABA for different durations in order to investigate the impact of the hormone on the capacity of seeds to respond to SL. Germination rate, *PrCYP707A1* expression, and minimal conditioning time were then investigated. The expected minimum conditioning period of 4 days in IM was observed (Fig. 1A) as already reported by Lechat *et al.* (2012). However, when incubated in IM supplemented with 250 µM ABA and then treated with 1nM GR24, seeds did not germinate after 4 or 7 days of conditioning (5±2% and 7±5%), in contrast to seeds incubated in ABA-free IM (74±6% and 86±6%) (Fig. 1A). To further determine whether ABA controlled the length of the conditioning period, seeds conditioned for 7 days in 250 µM of ABA were washed and then subsequently stimulated with 1nM GR24. In those conditions, 77±6% of seeds had germinated 3 days after stimulation, which was not statistically different from the germination rate of seeds incubated in ABA-free IM. Therefore, ABA did not prevent seed germination when applied only during the conditioning period. In parallel, *PrCYP707A1* expression was evaluated in seeds conditioned or not in 1mM ABA and then treated for 6h with 1nM GR24 without removing ABA. In these conditions, while seed germination was inhibited (Fig. 1A), GR24-induced up-regulation of *PrCYP707A1* was not affected after the minimal conditioning time (Fig. 1B). Altogether, these results demonstrate that although an exogenous ABA treatment unambiguously inhibited *P. ramosa* germination, it did not control the minimal conditioning period because up-regulation of *PrCYP707A1* still occurred in response to SL. This indicates that ABA does not influence the capacity of seeds to respond to SL.

### Global DNA demethylation during the conditioning period

**Fig. 2.**
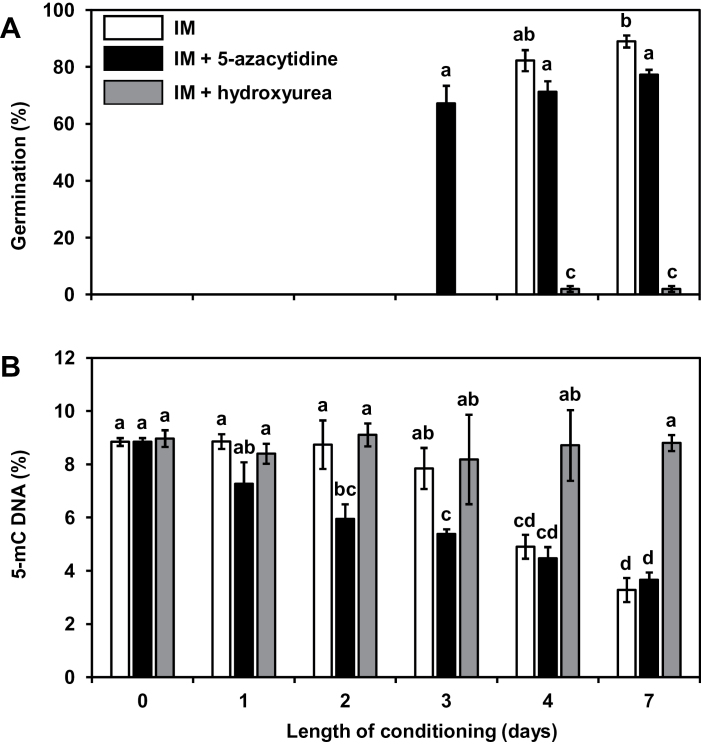
A global DNA demethylation occurs during the conditioning period. **(A)** Germination rate of *P. ramosa* seeds conditioned in IM supplemented or not with 250 µM 5-azacytidine or 250 µM hydroxyurea. After different lengths of conditioning, seeds were stimulated by 1nM GR24 and germination rates were determined after 3 days. Means are values ± SD (n = 6). **(B)** Percentage of 5-mC in seeds conditioned for different lengths of conditioning in IM supplemented (black bars) or not with 1mM 5-azacytidine (white bars) or 1mM hydroxyurea (grey bars). Means are values ± SD (n = 3). Means with the same letter are not significantly different from each other (Tukey test, *P* < 0.05).

The evolution of the global DNA methylation level in the seeds during the conditioning period was estimated and compared to the germination kinetic in response to GR24 (Fig. 2). To this end, the percentage of methylated DNA, corresponding to the proportion of 5-mC in DNA, was evaluated each day of the conditioning period using a global DNA methylation quantification method. When seeds were conditioned in IM, the minimum period required for the seeds to germinate in response to GR24 was, as expected, 4 days (82±7%) (Fig. 2A). In these conditions, DNA methylation level remained stable (8.9±0.1%) during the first 2 days of conditioning, and then significantly decreased from day 3 onwards (7.8±0.7%) (Fig. 2B) to reach nearly half of the initial level (4.9±0.5%) on day 4, when seeds gained the ability to germinate in response to GR24. The DNA methylation level then remained stable at a low value (3.3±0.8%) during the following days. Interestingly, when seeds were conditioned in ABA-containing IM for 7 days, the DNA methylation level also decreased to reach a value representing about half of the initial level (4.1±0.3%).

To determine if the observed DNA demethylation process occurring in *P. ramosa* during conditioning has a key role in the acquisition of the ability to germinate in response to SL, 250 µM 5-azacytidine, a hypomethylating agent, was added to the IM from the first day of the conditioning period and maintained throughout the experiment even after GR24 treatment. In these conditions, the minimum conditioning period required to reach the optimal germination rate upon GR24 treatment was reduced to 3 days (67±6%) instead of 4 days in control conditions (82±7%) (Fig. 2A). A longer conditioning period in the presence of 5-azacytidine did not significantly affect the maximum germination rate (Fig. 2A). In the meantime, the evolution of the methylation status during the conditioning period was determined and compared between control and 5-azacytidine conditions (Fig. 2B). In comparison with untreated seeds, DNA methylation levels in seeds treated with 5-azacytidine decreased continuously and regularly from 8.9±0.3% on day 1 just after imbibition to 5.3±0.2% on day 3, when germination could be triggered by GR24 treatment. The DNA methylation level finally reached the low value of 3.7±0.3%, which was similar to the level observed in untreated seeds, after 7 days.

**Fig. 3.**
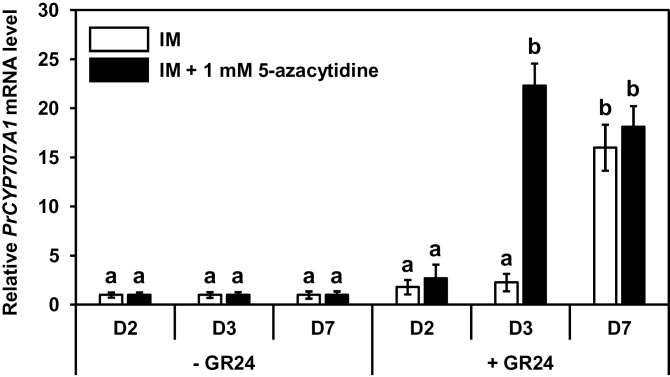
Global DNA methylation controls *PrCYP707A1* expression. RT-PCR analysis of the *PrCYP707A1* expression in seeds conditioned 2 (D2), 3 (D3), or 7 (D7) days in IM supplemented or not with 1mM 5-azacytidine. After conditioning, seeds were stimulated by 1nM GR24 (+GR24) or not (−GR24) and expression levels were determined after 6h. Means are values ± SD (n = 3). Means with the same letter are not significantly different from each other (Tukey test, *P* < 0.05).

Using real-time RT-PCR assays, the *PrCYP707A1* expression level was assessed in seeds conditioned 2, 3, and 7 days in IM supplemented or not with 1mM 5-azacytidine followed by 6h treatment with 1nM GR24 (Fig. 3). After 2 and 3 days of conditioning in IM, GR24 treatment did not trigger either *PrCYP707A1* overexpression nor seed germination; after a 7-day conditioning period, GR24 induced *PrCYP707A1* expression and subsequent seed germination. By contrast, when seeds were conditioned in IM containing 5-azacytidine, GR24 treatment triggered *PrCYP707A1* overexpression (relative expression, 24.3±2.3) one day earlier, i.e. after 3 days of conditioning. Seeds conditioned in 5-azacytidine for 7 days displayed *PrCYP707A1* expression levels upon GR24 stimulation (18.1±2.1) that are similar to those observed in untreated seeds (16.1±2.3). Thus, sensitivity to GR24 and *PrCYP707A1* inducibility seemed to depend on a fine-tuned global DNA methylation level. Indeed, *PrCYP707A1* expression was triggered upon GR24 treatment after 3 days in 5-azacytidine-containing IM or after 4 days in 5-azacytidine-free IM, when DNA methylation levels were statistically equivalent (with 5-azacytidine: 5.3±0.2%; without 5-azacytidine: 4.9±0.5%). After 2 days in 5-azacytidine-containing IM, *PrCYP707A1* was not induced, when the DNA methylation level was not significantly different (5.9±0.5%), suggesting that a threshold value of DNA methylation should be reached. Under this threshold value, which is independent of ABA level, and according to an on–off response, seeds are then receptive to GR24, *PrCYP707A1* is up-regulated, and seeds germinate.

### Impact of global DNA hypermethylation on *PrCYP707A1* expression and seed germination in response to GR24

**Fig. 4.**
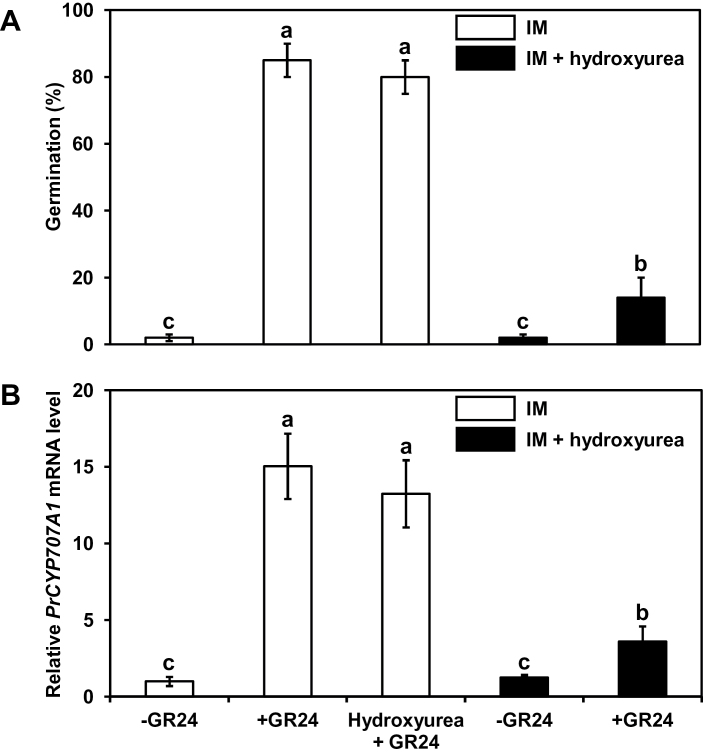
The hypermethylating compound hydroxyurea applied during the conditioning period inhibits GR24-dependent up-regulation of *PrCYP707A1* and subsequent germination. **(A)** Germination rates of *P. ramosa* seeds conditioned in IM supplemented or not with 250 µM hydroxyurea. After 7 days of conditioning, seeds were stimulated by 1nM GR24 (+GR24) or not (−GR24) and germination rates were determined after 3 days. Means are values ± SD (n = 6). **(B)** RT-PCR analysis of the expression of *PrCYP707A1* in seeds conditioned 7 days in IM supplemented or not with 1mM hydroxyurea. After conditioning, seeds were stimulated (+GR24) or not (−GR24) by 1nM GR24 treatment and expression levels were determined after 6h. Means are values ± SD (n = 3). Means with the same letter are not significantly different from each other (Tukey test, *P* < 0.05). “Hydroxyurea + GR24” corresponds to a treatment with both compounds after conditioning.

To further investigate the requirement of a change in DNA methylation status prior to seed germination, hydroxyurea, a hypermethylating agent, was applied during seed conditioning (Fig. 4). While GR24-treated seeds conditioned for 7 days in IM showed a high germination rate (86±4.2%), GR24-treated seeds incubated for 7 days in IM containing hydroxyurea exhibited lower germination rates (13±6.0%). Seeds conditioned for 7 days in IM then concomitantly treated with GR24 and hydroxyurea did not display any reduction in the optimum germination rate (81±4.8%), which was statistically equivalent to that observed in GR24 only-treated seeds (Fig. 4A). Together, these data suggested that hydroxyurea did not affect the germination process itself but rather the completion of the conditioning period.

RT-PCR assays showed that *PrCYP707A1* expression in seeds conditioned for 7 days in IM was strongly induced upon GR24 treatment (15±2.1) (Fig. 4B). A concomitant treatment with GR24 and hydroxyurea did not induce any significant modification in the gene up-regulation. By contrast, when hydroxyurea was applied during conditioning, an almost 4-fold reduction in *PrCYP707A1* transcript levels was observed in comparison to untreated seeds. This change came with a low germination rate (Fig. 4A). In addition, hydroxyurea treatment prevented a reduction in DNA methylation level, which remained stable at 8.7±0.4% throughout the conditioning period (Fig. 2B). Treatment was reversible and not lethal. Indeed, when seeds treated with hydroxyurea during conditioning were washed and conditioned for a second 7-day period, a germination rate of 67±7.9% was observed.

Thus, hypermethylating treatment prevented the seeds from responding to GR24 and germinating by maintaining DNA methylation levels above the determined threshold value. Acquisition of GR24 responsiveness is therefore under the control of a DNA demethylation process during the conditioning period.

### DNA demethylation in the *PrCYP707A1* promoter during conditioning

**Fig. 5.**
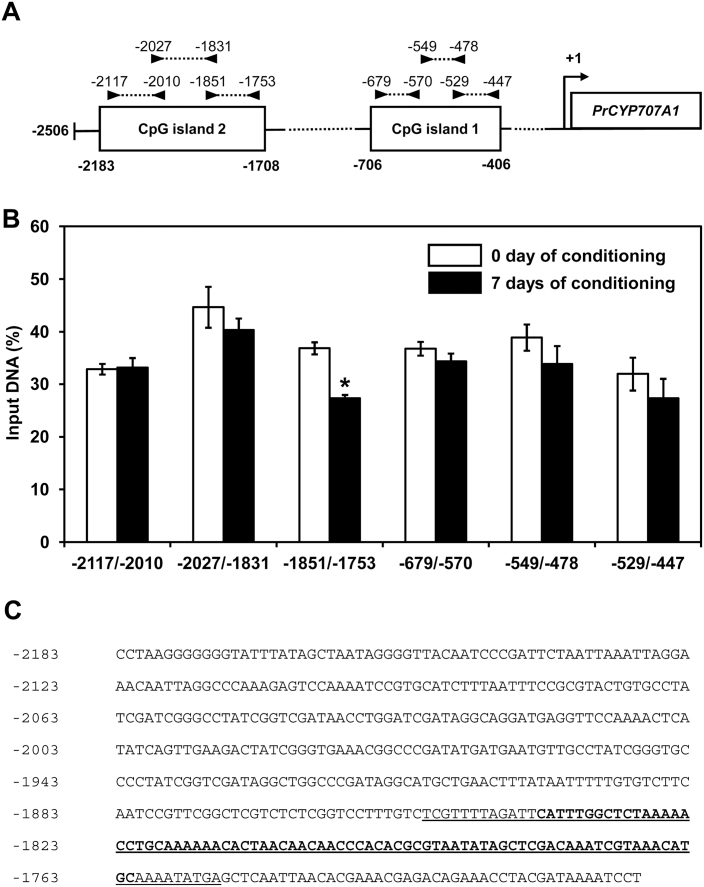
DNA demethylation occurs in the promoter of *PrCYP707A1*. **(A)** Localization of CpG islands and primer sets (black arrow) used in MeDIP-PCR on the *PrCYP707A1* promoter. Values correspond to island and primer positions in relation to the +1 transcription start site. **(B)** Methylation status of CpG islands on the *PrCYP707A1* promoter assayed by MeDIP-PCR. Expression values after 0 or 7 days of conditioning were normalized with non-immunoprecipitated DNA. Values are percentages of immunoprecipitated DNA compared to initial DNA quantity used in the immunoprecipitation assay (input DNA %). Values are means ± SD (n = 3). An asterisk indicates a significant methylation difference between 0 and 7 days of conditioning (Tukey test, *P* < 0.05). **(C)** Nucleotide sequence of CpG island 2. Underlined nucleotides correspond to the −1851/−1753 region exhibiting a DNA demethylation as shown by MeDIP-PCR. Nucleotides in bold correspond to the −1839/−1762 sequence mostly demethylated during conditioning as demonstrated by bisulfite sequencing.

To determine if the global DNA demethylation process observed during the conditioning period might also occur at the nucleotide level in the *PrCYP707A1* promoter sequence, putative methylated sites were analysed (Fig. 5). First, the *PrCYP707A1* promoter sequence was obtained using a chromosome walking strategy (Fig. 5A). Then, *in silico* analyses were used to locate CpG islands. Only two CpG islands were found, at −2183/−1708bp and −706/−406bp from the transcription start site. The methylation status of these two CpG islands was examined by MeDIP-PCR assay on day 0 and day 7 of the conditioning period. To this end, purified DNA from the immunoprecipitated DNA complexes and from input DNA was analysed by quantitative RT-PCR. Sequences of both CpG islands were amplified using three primer sets covering 78% and 72% of the sequences, respectively (Fig. 5A). The relative changes in the extent of sequence methylation were determined by measuring the amount of detected sequence in immunoprecipitated DNA after normalization to the input DNA (Fig. 5B). While CpG island 1 exhibited no change in methylation rate during the conditioning period, a significant demethylation was observed in CpG island 2. Indeed, in CpG island 2, the methylation rate of the region located between −1851 and −1753 (Fig. 5C) was 36.9±1.1% of input DNA at the beginning of conditioning and significantly decreased to 27.3±0.6% after 7 days of conditioning (Fig. 5B).

**Fig. 6.**
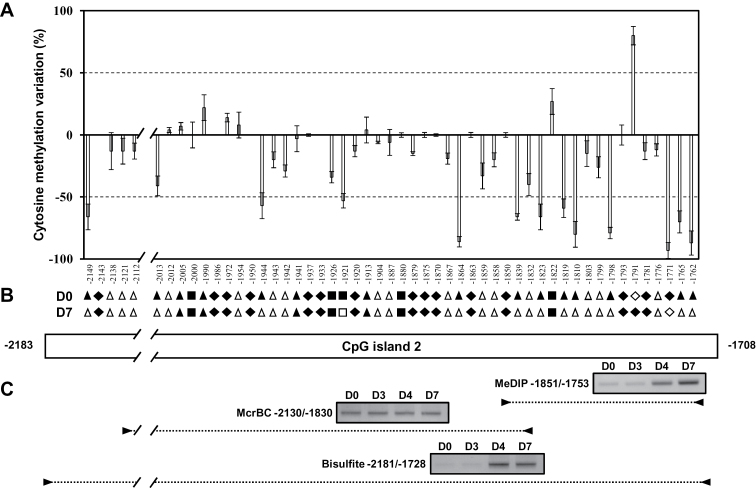
DNA demethylation occurs in a 78-bp region of the *PrCYP707A1* promoter. **(A)** Cytosine methylation variations of the CpG island 2 in the *PrCYP707A1* promoter during seed conditioning assayed by bisulfite sequencing. DNA from D0 and D7 seeds were treated with bisulfite and the CpG island 2 region was amplified. PCR products were cloned and a total of 20 clones per condition were sequenced. Negative numbers in the x-axis indicate cytosine position from the transcription start site. For a given cytosine, a vertical bar corresponds to the cytosine methylation variation, expressed as a percentage, calculated according to the following formula: (number of clones showing a methylated cytosine at D7 minus the number of clones showing a methylated cytosine at D0) divided by 20. Thus, a positive value indicates a cytosine methylation process, a negative value a cytosine demethylation process, and a null value no variation in the methylation status during the conditioning period. **(B)** Detailed methylation profiles for CpG island 2 at 0 (D0) and 7 days (D7) of conditioning. Potentially methylated sites CG, CHG, and asymmetric CHH (H = A, T, or C) are shown by diamonds, squares, and triangles, respectively. According to their methylation percentages, cytosine sites are considered as hypermethylated (% of methylated cytosine > 50%, filled symbols), or hypomethylated (% of methylated cytosine < 50%, open symbols) (Trap-Gentil *et al.*, 2011). **(C)** CG methylation status of CpG island 2 at 0 (D0), 3 (D3), 4 (D4), and 7 days (D7) of conditioning assayed by MS-PCR and three primer sets (black arrow). The *McrBC* enzyme cuts in hypermethylated regions.

To investigate more accurately this DNA demethylation process, a bisulfite sequencing experiment was performed (Fig. 6), in which a primer set was designed (Bisulfite −2181/−1728) to amplify most of CpG island 2 following bisulfite treatment. A 78-bp region (−1839/−1762) in this 475 nucleotide sequence showed statistically significant DNA demethylation (Figs 5C and 6A), corroborating the result obtained by MeDIP-PCR (Fig. 5B) and thus confirming that the −1851/−1753 region of CpG island 2 was the main sequence epigenetically impacted. Indeed, following Trap-Gentil *et al.* (2011), this sequence (−1839/−1762) was determined to be hypermethylated at D0 (68.7% of 5-mC) and hypomethylated at D7 (25% of 5-mC), with the CHH motif the most affected (63.6% methylated at D0 and 0% at D7) (Fig. 6B).

Finally, all these results were confirmed by MS-PCR using *McrBC* restriction enzyme and three primer sets, one corresponding to the complete CpG island 2 (Bisulfite −2181/−1728) and two partially covering this island (McrBC −2130/−1830 and MeDIP−1851/−1753) (Fig. 6C). The amplification of the McrBC −2130/−1830 sequence was not affected by *McrBC* restriction whereas the Bisulfite −2181/−1728 and MeDIP −1851/−1753 sequences showed the strongest amplification at D4 and D7, suggesting that the shared −1851/−1753 sequence was less methylated at D4 and D7 than at D3 and D0. These results confirmed the hypomethylation of the −1839/−1762 region determined by MeDIP-PCR and bisulfite sequencing approaches.

Taken together, these results demonstrate that a 78-bp region of CpG island 2 localized in the *PrCYP707A1* promoter undergoes a DNA demethylation process during the conditioning period.

## Discussion

Seed germination of obligate root parasitic plants from the Orobanchaceae family, including species of the *Orobanche* and *Phelipanche* or *Striga* genus, requires stimulation by host-derived GS (Xie *et al.*, 2010). Recently, seed germination in *P*. *ramosa* in response to the synthetic SL GR24 was shown to be mediated by a strong up-regulation of *PrCYP707A1*, an ABA catabolic gene, after a minimal conditioning period (Lechat *et al.*, 2012). Although it was initially thought that the acquisition of sensitivity to SL during the conditioning period might be controlled by endogenous ABA levels, the results from this study showed that, when ABA was supplied during the conditioning period, *PrCYP707A1* up-regulation was still triggered upon GR24 treatment. This finding indicates that the minimal period required for the seeds to respond to SL, referred to as the conditioning period, is not controlled by endogenous ABA content.

Recent studies suggest that some hormone-independent mechanisms impacting gene expression may also occur during dormancy and germination in plants (Nonogaki, 2014). These are mainly epigenetic mechanisms, such as histone deacetylation and ubiquitination, and histone and DNA methylation (Hsieh and Fischer, 2005; van Zanten *et al.*, 2013). DNA methylation represents an important epigenetic mechanism that influences chromatin structure and gene regulation, the modulation of which plays a major role in the regulation of plant development, notably seed germination in response to environmental conditions (Portis *et al.*, 2004). The distribution and the number of 5-mC on DNA molecule is heritable but can also vary according to the developmental stage (Koukalova *et al.*, 2005). Thus, a global DNA demethylation process was observed during seed imbibition in pepper, rapeseed, and wheat (Portis *et al.*, 2004; Lu *et al.*, 2006; Meng *et al.*, 2012). Nevertheless, the roles of this DNA demethylation within the germination process remain unclear.

In the present study, a global DNA methylation quantification method was used to demonstrate that the total DNA methylation level decreased in a discontinuous manner in *P. ramosa* seeds during the conditioning period, independently of ABA. Indeed, this decrease was not impacted by ABA treatment and occurred between the third and fourth day, after which seed germination could be triggered by GR24. When demethylation was artificially generated by applying the hypomethylating compound 5-azacytidine during the conditioning period, methylation levels similar to those assessed after 4 days in natural conditions were achieved only after 3 days. In the meantime, *PrCYP707A1* expression was up-regulated following GR24 treatment after 3 days with the demethylating compound or 4 days without. Seed ability to germinate therefore happened 1 day earlier in hypomethylated seeds. Taken together, these results suggest that *P. ramosa* seeds can germinate, following GR24-dependent *PrCYP707A1* activation, only when global DNA methylation reaches a sufficiently low level. In agreement with that, a DNA hypermethylating treatment with hydroxyurea during the conditioning phase maintained a high and constant DNA methylation level in conditioned seeds, preventing *PrCYP707A1* induction by GR24 and subsequent seed germination. Concordant results have also been reported in a number of autotrophic plants, showing, for instance, higher methylation levels in dry seeds than in tissues from germinating seeds (Portis *et al.*, 2004; Lu *et al.*, 2006; Meng *et al.*, 2012). Similarly, a demethylation process occurring mainly in the endosperm tissue was also demonstrated during the first hour of water imbibition in seeds of *Silene latifolia* (Zluvova *et al.*, 2001). Interestingly, the *Arabidopsis DML3* gene encodes a DEMETER-like protein by removing DNA methylation marks from improperly methylated cytosines, as well as being involved in the maintenance of high DNA methylation levels in properly targeted sites (Ortega-Galisteo *et al.*, 2008). Kim *et al.* (2010) demonstrated that acceleration in seed germination under stress conditions was accompanied by DML3 mRNA degradation as a consequence of the microRNA miR402 overexpression.

The above findings suggest that seed germination can be prevented by high methylation levels of cytosine in specific targeted sites. In this scheme, dormancy release in *P. ramosa* seeds would consist of a two-step process, starting with the conditioning period characterized by a global DNA demethylation required for the establishment of the second phase, which corresponds to a GR24-dependent ABA catabolism. Among the actors involved in the response to GR24, *PrCYP707A1* was shown to be up-regulated only after a sufficiently low 5-mC level was achieved, suggesting that the expression of this gene was affected by this process and could thus be a key component of seed germination. Interestingly, in potato tuber meristems, bromoethane treatments induce a significant transient decrease in the level of 5-mC, leading to a chemically forced dormancy release (Law and Suttle, 2003). Bromoethane treatment also induces a strong up-regulation of three potato *CYP707A* genes, explaining the dormancy breakdown related to an enhanced ABA catabolism (Destefano-Beltrán *et al.*, 2006). These results indicate, therefore, that expression of *CYP707A* genes in potato tuber could be under the control of epigenetic mechanisms such as DNA methylation.

In the present study, using complementary MeDIP, MS-PCR, and bisulfite sequencing approaches, the DNA methylation status of the *PrCYP707A1* promoter was investigated at the nucleotide level during the conditioning period. Two CpG islands were identified by *in silico* analysis, out of which only island 2 showed significant demethylation of cytosine residues, all of them being mainly localized within a 78-nucleotide region. This site-specific decrease in 5-mC level is likely to permit SL-dependent gene overexpression and then ABA catabolizing seed germination. However, *in silico* analysis of the *PrCYP707A1* promoter using PLACE software (Higo *et al.*, 1999) did not reveal any known cis-regulating sequence related to the seed germination process in the 78-nucleotide hypomethylated region (data not shown). Although the results presented here show unambiguously that demethylation of a specific region of the *PrCYP707A1* promoter is required for SL-dependent gene expression, this variation is not sufficient to explain the observed decrease in global DNA methylation during the conditioning period. This therefore suggests that this epigenetic process may also target other molecular actors.

The observed modifications in DNA methylation levels may result from tissue-specific effects (Meng *et al.*, 2012). Indeed, tissues surrounding the embryo were shown to be more intensively demethylated than the embryo during seed imbibition in wheat and oilseed rape (Lu *et al.*, 2006; Meng *et al.*, 2012). In addition, extensive DNA demethylation in the endosperms of *Arabidopsis* (Hsieh *et al.*, 2009), *Oryza sativa* (rice) (Zemach *et al.*, 2010), and *Zea mays* (maize) (Lauria *et al.*, 2004) and local histone modifications in *Brachypodium distachyon* embryos (Wolny *et al.*, 2014) have been reported. Interestingly, in *P. ramosa* seeds, *PrCYP707A1* was shown to be expressed in the perisperm cells close to the micropyle a few hours after GR24 stimulation (Lechat *et al.*, 2012), suggesting a spatial distribution of epigenetic modifications during seed germination in *P. ramosa*, as already demonstrated in autotrophic plants.

DNA demethylation may take place as a passive process due to a lack of methylation maintenance during several cycles of DNA replication, or as an active mechanism (in the absence of replication) mediated by DNA glycosylase domain-containing proteins that have the capacity to erase 5-mC (Roldán-Arjona and Ariza, 2009). Interestingly, DNA demethylation in *P. ramosa* seeds during the conditioning period is not a continuous and progressive process because a significant decrease in 5-mC methylation only occurred after 3 days. This suggests that an active mechanism could be triggered a few days after seed imbibition. In the genetically close species *P. aegyptiaca*, Mayer and Bar-Nun (1993) demonstrated a steady rate of thymidine incorporation during conditioning without any change in total DNA, indicating that incorporation was not due to any DNA replicative synthesis. In *S. latifolia*, DNA demethylation that occurred during germination took place in a non-replicative process (Zluvova *et al.*, 2001). Although this point needs to be confirmed in the future, it might then be suggested that the DNA demethylation observed during *P. ramosa* conditioning was not due to replication but rather to active mechanisms.

Although experimental evidences from the present study unequivocally established the importance of DNA methylation during seed germination in *P. ramosa*, other epigenetic modifications such as histone acetylation and/or methylation should, however, not be excluded and deserve to be further investigated. For instance, histone acetylation regulates seed dormancy in *Arabidopsis* by controlling expression of two ABA catabolic genes, *AtCYP707A1* and *AtCYP707A2* (Wang *et al.*, 2013). Indeed, global chromatin dynamics of some key regulatory genes have been shown to play major roles during seed life (Zhang and Ogas, 2009; Müller *et al.*, 2012). However, if DNA methylation is considered as a modulator of chromatin structure that also influences transcriptional regulation (Reddington *et al.*, 2013), inhibition of gene expression by DNA methylation is mainly known to be achieved through two general mechanisms: modification of cytosine that can hamper the binding of transcriptional machinery (RNA polymerase II and transcription factors) to important promoter DNA sequences (Watt and Molloy, 1988); and repressing protein complexes that specifically recognize methylated DNA (Boyes and Bird, 1991).

In the case of *PrCYP707A1*, whatever the involved mechanisms, while gene silencing should be released by a DNA demethylation process, triggering of expression still requires GS. The question to address would now be whether demethylation and GS stimulation are independent or closely linked processes. Several studies on shoot branching and root development have led to the definition of a model concerning the SL signalling pathway. In this model, SL are bound to and hydrolysed by the α/β hydrolase protein D14 and interact with the nuclear localized F-box protein MORE AXILLARY GROWTH 2 (MAX2). This complex is thought to select candidate proteins for ubiquitination and subsequent degradation by the 26S proteasome (de Saint Germain *et al.*, 2013). Recently, a few of these proteasome target proteins have been identified in rice (Zhou *et al.*, 2013) and *Arabidopsis* (Stanga *et al.*, 2013; Wang *et al.*, 2013), and may act as transcription repressors. In this scheme, degradation of these repressors would trigger downstream signalling events via gene up-regulation. Although the SL signalling pathway involved in the seed germination of parasitic plants has not yet been elucidated, some preliminary results implicate the α/β hydrolase/MAX2 complex in this process. First, it has been clearly demonstrated that at least one *P. aegyptiaca KAI2* paralog, encoding an α/β hydrolase homologous to D14, could restore the sensitivity of *Arabidopsis kai2* mutant seeds to GR24 (C.E. Conn, D. Neumann, K.A. Dyer and D.C. Nelson, unpublished). Second, when the proteasome inhibitor MG132 is applied together with GR24 to *P. ramosa* conditioned seeds, it prevents *PrCYP707A1* up-regulation and subsequent seed germination (unpublished data). Thus, it can be hypothesized that DNA methylation along with other epigenetic marks such as histone modifications may influence chromatin structure, thereby rendering inaccessible a component of *PrCYP707A1* expression to degradation by KAI2/MAX2 activity.

Regarding the *P. ramosa* life cycle, the *raison d’être* of such a fine-tuned process might, therefore, be to afford the seeds the necessary time to set up germination and to prevent premature activation of *PrCYP707A1*-dependent ABA catabolism by SL. Such a mechanism would thus be of major interest for the reproductive success of this parasitic plant. Given the alarming impact of broomrape species on world agriculture, deciphering their developmental and metabolic particularities is a necessary step toward the development of targeted control methods. Thus, every physiological and molecular event governing germination, such as DNA methylation, can be considered a point of vulnerability that could potentially be exploited. Indeed, control of these parasitic weeds can be achieved by preventing seed germination through biocontrol agents or, in contrast, by promoting seed germination in the absence of host plants in order to reduce the seed bank of the soils. At a more fundamental level, the results presented here demonstrate that DNA methylation, known to be active during seed germination in autotrophic plants, is also involved in the particular response of root parasitic plant seeds to SL. Whether an epigenetic control also influences the known SL responses of autotrophic plants by modulating shoot branching and root growth nevertheless still awaits investigation.

## Abbreviations:

5-mC: 5-methylcytosine
ABA: abscisic acid
GS: germination stimulant
IM: incubation medium
IP: immunoprecipitation
MeDIP: methylated DNA immunoprecipitation
MS-PCR: methylation-sensitive polymerase chain reaction
SL: strigolactone.
